# Bone Marrow Adiposity Alterations in Postmenopausal Women With Type 2 Diabetes Are Site-Specific

**DOI:** 10.1210/jendso/bvae161

**Published:** 2024-09-13

**Authors:** Sammy Badr, Anne Cotten, Daniela Lombardo, Stefan Ruschke, Dimitrios C Karampinos, Nassima Ramdane, Michael Genin, Julien Paccou

**Affiliations:** Department of Radiology and Musculoskeletal Imaging, University Lille, CHU Lille, MABlab ULR 4490, F-59000 Lille, France; Department of Radiology and Musculoskeletal Imaging, University Lille, CHU Lille, MABlab ULR 4490, F-59000 Lille, France; Department of Rheumatology, CHU Lille, F-59000 Lille, France; Department of Diagnostic and Interventional Radiology, Klinikum rechts der Isar, School of Medicine and Health, Technical University of Munich, 81675 Munich, Germany; Department of Diagnostic and Interventional Radiology, Klinikum rechts der Isar, School of Medicine and Health, Technical University of Munich, 81675 Munich, Germany; Department of Biostatistics, CHU Lille, F-59000 Lille, France; ULR 2694—METRICS: Évaluation des Technologies de Santé et des Pratiques Médicales, University Lille, CHU Lille, F-59000 Lille, France; Department of Rheumatology, University Lille, CHU Lille, MABlab ULR 4490, F-59000 Lille, France

**Keywords:** bone marrow adipose tissue, diabetes mellitus, glycated hemoglobin, bone mineral density, osteoporosis

## Abstract

**Context:**

Bone marrow adiposity (BMAT) alterations in patients with type 2 diabetes mellitus (T2DM) may contribute to adverse bone effects.

**Objective:**

Characterization of BMAT content and composition in patients with well-controlled T2DM.

**Methods:**

This cross-sectional study included 2 groups of postmenopausal women: one with T2DM and the other without. The proton density fat fraction (PDFF) of the lumbar spine and proximal femur, comprising the femoral head, neck, and diaphysis, was assessed using chemical shift-based water-fat separation imaging (WFI). Magnetic resonance imaging with spectroscopy (^1^H-MRS) was performed in a subgroup of participants to confirm the PDFF measurements and determine the apparent lipid unsaturation level (aLUL) at the L3 vertebrae and femoral neck. The association of imaging-based PDFFs and aLUL between diabetes groups was investigated by adjusting for confounding factors using a linear mixed model.

**Results:**

Among 199 participants, patients with T2DM (n = 29) were significantly heavier (*P* < .001) and had a higher bone mineral density (BMD) (*P* < .001 for all sites) than nondiabetic patients (n = 170). When PDFFs were compared after adjusting for age, body mass index (BMI), and BMD, the femoral head WFI-based PDFF was lower in patients with T2DM (mean [standard error] 88.0% [0.7] vs 90.6% [0.3], *P* < .001). Moreover, the aLUL at the L3 vertebrae was lower in patients with T2DM (n = 16) than in without (n = 97) (mean [standard error] 3.9% [0.1] vs 4.3% [0.1], *P* = .02).

**Conclusion:**

The content and composition of BMAT are modified in postmenopausal women with T2DM and these changes occur at specific sites.

The musculoskeletal system has received less attention as a target of organ damage in diabetes; however, it is now recognized that increased bone fragility is a common and severe complication of the disease, particularly in older individuals with multiple risk factors for falls and fractures [1, 2]. Changes in bone metabolism in patients with type 2 diabetes mellitus (T2DM) have been the subject of numerous studies [3, 4]. Type 2 diabetes mellitus is associated with normal/high bone mineral density (BMD), but low bone turnover, reduced bone strength, and increased fracture risk [5, 6]. Research has identified a variety of alterations at the matrix, cellular, and structural levels that might account for reduced bone strength despite normal/high BMD [7, 8].

While the mechanisms underlying bone alterations in patients with T2DM remain incompletely understood, multiple factors are involved, including hyperglycemia, oxidative stress, release of inflammatory factors and adipokines from visceral fat, and accumulation of advanced glycation end products (AGEs) [7, 9]. It has been postulated that changes in the bone marrow adipose tissue (BMAT) in patients with T2DM may contribute to adverse bone effects [10, 11]. Indeed, recent bone histomorphometry data suggest that increased BMAT levels occur through adipocyte hyperplasia and hypertrophy in premenopausal women with T2DM [12]. However, in contrast to animal studies that have clearly demonstrated higher levels of BMAT in diabetes [13, 14], magnetic resonance imaging (MRI) with spectroscopy (^1^H-MRS) studies of the lumbar spine in humans have shown smaller and less certain differences [15-18]. The most convincing data showed lower unsaturation levels at the lumbar spine in subjects with T2DM [15, 16].

Further investigation is necessary to corroborate these findings and to evaluate whether glycated hemoglobin (HbA1c), as a biomarker of glycemic control, is correlated with BMAT, as suggested by some authors [12, 15]. It is important to note that previous studies were limited by the small number of participants with T2DM and inclusion of women and men of all ages. Moreover, very few studies have investigated BMAT in the proximal femur [18].

We conducted this cross-sectional study using data from the ADIMOS cohort [19], with the main objective of characterizing the content and composition of BMAT in postmenopausal women with T2DM. In addition, we estimated the percentage of association between BMAT and T2DM mediated by the presence of a recent fracture and BMD (lumbar spine and femoral neck). Finally, the correlations between HbA1c and proton density fat fraction (PDFF) was evaluated.

## Methods

### Study Protocol

The ADIMOS study is a case-control investigation (NCT03219125) aimed at exploring the association between imaging-based PDFF and fragility fractures in postmenopausal women at the lumbar spine and proximal femur [19]. The subjects selected were postmenopausal women with recent fragility fractures (less than 12 months ago) as cases and postmenopausal women with osteoarthritis and no history of fragility fractures as controls. Chronic kidney disease with a calculated creatinine clearance of less than 30 mL/min/1.73 cm^2^ and contraindications to MRI were among the exclusion criteria. Additionally, the current use of substances known to affect BMAT, including glucocorticoids, osteoporosis medications (bisphosphonates, raloxifene, calcitonin, or teriparatide), thiazolidinediones, and estrogen replacement therapy, was also excluded. However, prior use of osteoporosis medications and estrogen replacement therapy over 12 months of age was allowed.

This ancillary ADIMOS-T2DM study was approved by the local Institutional Review Board. All participants provided written informed consent before participation.

### Study Participants

Postmenopausal women aged 45 to 95 years, with and without T2DM, were recruited from the University of Lille. Type 2 diabetes mellitus diagnosis was defined as either a self-reported diagnosis, receipt of pharmacological treatment, or an HbA1c level ≥ 6.5%.

The following risk factors for osteoporosis have been documented: current smoking status, heavy alcohol consumption, and prior use of oral corticosteroids. Additionally, information was gathered on the Charlson Comorbidity Index (CCI), leisure time activities (scored on a scale of 0-15), and medication usage.

### Imaging-Based Proton Density Fat Fraction

MRI examinations were performed using a 3-T full-body scanner (Ingenia; Philips Medical Systems, Best, The Netherlands), which featured a built-in 12-channel posterior body coil and 16-channel anterior coil. The patients were placed in the supine position and introduced head-first into the scanner tunnel. The imaging protocol was overseen by a senior musculoskeletal radiologist and comprised both conventional exploration of the lumbar spine for morphological assessment and optimized chemical shift encoding-based water-fat imaging (WFI) sequences (mDixon-Quant; Philips Healthcare, Best, The Netherlands) at the lumbar spine and nondominant hip for BMAT quantification. The PDFF maps were computed offline using a precalibrated 7-peak fat spectrum and a single T2* correction, without gadolinium contrast administration, as part of the protocol for acquiring MRI images to determine the PDFF.

MRI segmentation was carried out on a dedicated workstation using the IntelliSpace Portal software (Philips Healthcare, Best, Netherlands) for the purpose of conducting this study. Specifically, 3 of the most centrally located slices of the computed maps were selected and a polygonal region of interest was drawn around the vertebral bodies of the lumbar spine from L1 to L4, excluding the fractured vertebrae, immediate subchondral bone, bone marrow-replacing lesions, severe degenerative changes, and basivertebral veins. The average PDFF value for the lumbar spine was obtained for each participant by averaging the segmented vertebrae L1–L4. Similarly, the average PDFF value for the nondominant hip was obtained by drawing a region of interest around the femoral head, femoral neck, and proximal femoral diaphysis, and taking the average value.

The ^1^H-MRS voxels were positioned in the L3 vertebral body and at the femoral neck of the nondominant hip using orthogonal scout sections and avoiding the cortical bone. If the L3 vertebral body was fractured, the voxel was placed at the L2 level. Postprocessing was performed using ALFONSO (A versatile Formulation fOr N-dimensional Signal model fitting of MR spectroscopy data) scripts written in MATLAB, version R2022a (MathWorks) [20]. The fitting strategy used common T2 values and linewidth constraints across all 10 fat peaks [21]. PDFF was calculated as the percentage of the fat signal relative to the total signal intensity (fat + water). The apparent lipid unsaturation level (aLUL) was calculated using the olefinic peak (UL) as the most representative unsaturated lipid: aLUL (%) = UL/total fat.

As for WFI acquisitions, spectroscopic sequence parameters were described and published in the main ADIMOS study [19].

### Laboratory Measurements

Blood samples were collected from all patients following an overnight fast, and standard assays were conducted to determine total calcium, phosphorus, and hs-CRP levels. Intact parathormone (PTHi) levels were measured using chemiluminescent immunoassay on an automatic analyzer (Architect, Abbott Laboratories, USA). Levels of 25-OH vitamin D were determined using a competitive chemiluminescent immunoassay on an IDS-iSYS device (IDS, Pouilly en auxois, France). Procollagen I intact N-terminal (PINP) and serum cross-laps (CTX) were measured using a chemiluminescence assay on an IDS-iSYS Multi-Discipline Automated Analyzer (Immunodiagnostic Systems, Inc., Fountain Hills, AZ, USA).

### BMD Measurements

Bone mineral density was assessed at the lumbar spine (L1–L4) and nondominant hip using dual-energy x-ray absorptiometry (DXA; HOLOGIC Discovery A S/N 81360). The data collected comprised BMD measurements (in grams per square centimeter of hydroxyapatite) at 3 separate sites: the lumbar spine, total hip, and femoral neck. In accordance with the World Health Organization's guidelines, osteoporosis was diagnosed if the T-score was equal to or less than −2.5.

### Statistical Analyses

Categorical variables were expressed as numbers (percentages). Continuous variables were expressed as mean (SD) in the case of normal distribution or median [interquartile range]. Normality of distribution was assessed using histograms and the Shapiro-Wilk test.

Patient characteristics and biochemistry data were described and compared according to the presence or absence of diabetes using a Chi-square or Fisher exact test (when the validity conditions of the Chi-square test were not verified) for categorical variables, Mann-Whitney U test for non-Gaussian continuous variables, and Student *t* test for Gaussian continuous variables.

The association of imaging-based PDFF and aLUL by MRI with spectroscopy (^1^H-MRS) between diabetes groups was investigated by adjusting for predefined confounding factors (age, BMI, femoral neck BMD, or lumbar spine BMD) using a linear mixed model. For each model, the normality of the residuals was graphically checked. Adjusted mean ± standard error of the mean (SEM) was derived from the mixed model. The magnitude of between-group differences was quantified by calculating the absolute standardized differences (ASD) with a 95% CI. Absolute values of 0.2, 0.5, and 0.8 in standardized differences were interpreted as small, medium, and large differences, respectively.

**Figure 1.**
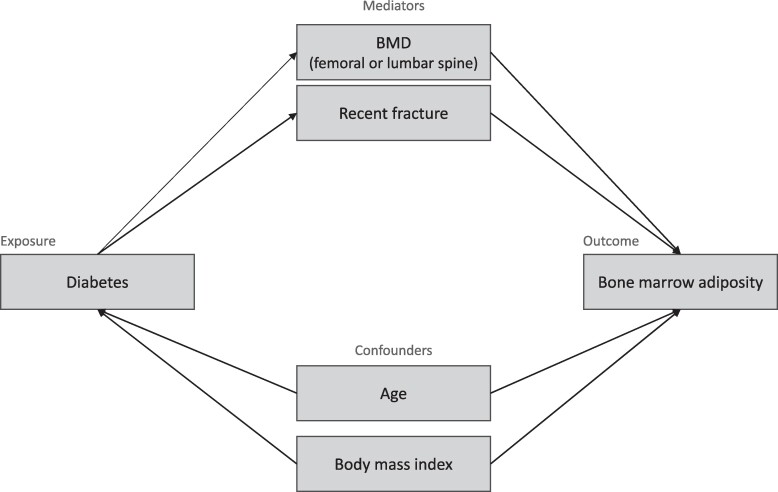
Direct acyclic graph of a structural model of mediation of the association between diabetes and bone marrow adiposity by the presence of a recent fracture and by BMD (lumbar spine or femoral neck).

To elucidate the association between the presence of diabetes and BMAT measurements (PDFF and aLUL), mediation analyses were performed to estimate indirect associations acting through the presence of a recent fracture, femoral neck BMD and lumbar spine BMD as mediating variables and direct associations not mediated by these latter variables. (Fig. 1). A parametric regression approach was used to estimate the total effect, natural indirect effects (NIE), and natural direct effects (NDE) of BMAT. For each BMAT measurement (PDFF and aLUL), 2 regression models were estimated: a multivariable logistic regression model for the presence of recent fractures conditional on the presence of diabetes and confounders (age and body mass index [BMI]) and a multivariable linear regression for the BMAT (outcome) conditional on the presence of T2DM, recent fracture, and all confounders. The same approach was used for femoral neck BMD and lumbar spine BMD, except that a multivariable linear regression model was used instead of the logistic model. To quantify the magnitude of mediation, we estimated the percentage of association mediated by the presence of a recent fracture ([NIE/(NIE + NDE)] × 100).

In the overall population, in each of the groups of patients with and without T2DM, we assessed the correlation between lumbar spine and hip imaging- and MRS-based PDFF, and the HbA1c parameter by calculating Pearson correlation coefficients or Spearman rank correlation coefficients for non-Gaussian distributions.

Statistical analysis was performed using a 2-tailed α level of .05. Data were analyzed using the SAS software package (version 9.4; SAS Institute, Cary, NC, USA).

## Results

### Baseline Characteristics

Table 1 presents the baseline characteristics and biochemical results. The study group included 199 postmenopausal women (mean age 67.5 ± 10.0 years, range 50-94 years), with 100 patients with at least one recent clinical fracture. The vast majority of participants was of White ethnic background (> 95%). Twenty-nine patients had T2DM (14.6%) and 179 had no T2DM. The diabetes medications included metformin (n = 11), sulfonylurea (n = 6), insulin (n = 5), glucagon-like peptide 1 receptor agonists (GLP-1RA) (n = 4), and gliptins (n = 3). None of the patients reported the use of thiazolidinediones and 10 patients had no diabetes medication. Patients with T2DM were significantly heavier (*P* < .001) than those without diabetes. The osteoporosis risk factors in both groups were comparable, including a history of recent fragility fractures. Charlson Comorbidity Index was higher in patients with T2DM (*P* < .001).

**Table 1.** Patients’ general characteristics and biochemistry results

	N	No diabetes (n = 170)	N	Diabetes (n = 29)	*P* value
Age, years	170	66 (59 to 73)	29	69 (63 to 76)	.26
Weight, kg	170	66 (57 to 76)	29	80 (69 to 96)	**<**.**001**
Height, cm	170	160.0 ± 6.5	29	161.2 ± 6.8	.34
BMI, kg/m^2^	170	25.7 (22.9 to 29.1)	29	31.2 (26.8 to 35.1)	**<**.**001**
Leisure time activity, score 0-15	169	9 (7 to 11)	27	7 (6 to 9)	.**003**
Prior use of HRT/estrogen	170	32 (18.8)	29	1 (3.5)	.**011**
COMORBIDITIES					
Nonmetastatic cancer	170	33 (19.4)	29	5 (17.2)	.78
Chronic pulmonary disease	170	12 (7.1)	29	2 (6.9)	1.00
Stroke or TIA	170	6 (3.5)	29	5 (17.2)	.**012**
Charlson Comorbidity Index	170	2 (2 to 4)	29	5 (3 to 6)	**<**.**001**
FRACTURES					
Recent fracture	170	85 (50.0)	29	15 (51.7)	.86
Family history of hip fracture	170	19 (11.2)	29	3 (10.3)	1.00
BIOCHEMISTRY RESULTS					
HbA1c, %	169	5.5 (5.3 to 5.7)	29	6.7 (6.3 to 7.1)	**<**.**001**
Glycemia, g/L	170	0.96 (0.87 to 1.03)	29	1.27 (1.06 to 1.50)	**<**.**001**
Hs-CRP, mg/L	170	3 (3 to 5)	29	3 (3 to 12)	.10
Calcium, mmol/L	170	2.38 ± 0.10	29	2.43 ± 0.12	.**027**
25(OH) vitamin D, ng/mL	170	29 (22 to 33)	29	24 (17 to 36)	.12
Serum PTH, pg/mL	170	44.0 (34.0 to 57.0)	29	42.0 (34.0 to 60.0)	.61
Creatinine, µmol/L	170	62 (53 to 71)	29	71 (62 to 80)	.082
Creatinine clearance (MDRD formula), mL/min	170	85.8 (74.5 to 95.5)	29	81.0 (60.1 to 92.0)	.06
BIOCHEMICAL MARKERS of BONE					
PINP, ng/mL	164	66.0 (48.5 to 79.5)	28	39.5 (27.5 to 65.0)	**<**.**001**
CTX, pmol/L	169	3354 (2413 to 4899)	29	1943 (1356 to 3609)	.**004**
BONE MINERAL DENSITY					
BMD lumbar spine, g/cm^2^	170	0.870 ± 0.154	28*^a^*	1.031 ± 0.239	.**002**
BMD total hip, g/cm^2^	167*^b^*	0.794 ± 0.133	29	0.916 ± 0.197	.**003**
BMD femoral neck, g/cm^2^	167*^b^*	0.668 ± 0.121	29	0.745 ± 0.176	.**022**
FAT CONTENT (imaging-based) DIXON					
PDFF lumbar spine, %	170	58 (52 to 65)	29	61 (53 to 68)	.17
PDFF femoral head, %	163*^c^*	91.6 (88.5 to 93.3)	29	88.5 (83.5 to 90.6)	**<**.**001**
PDFF femoral neck, %	163*^c^*	83.7 (77.8 to 88.7)	29	78.1 (72.0 to 84.7)	.**004**
PDFF femoral diaphysis, %	163*^c^*	82.1 (75.7 to 88.2)	29	78.0 (70.1 to 82.8)	.**012**
FAT CONTENT (imaging-based) ^1^H-MRS					
L3 PDFF, %	97	58.1 (49.5 to 63.6)	16	62.2 (47.0 to 66.6)	.32
L3 aLUL, %	97	4.3 (3.9 to 4.6)	16	4.0 (3.7 to 4.2)	.051
Femoral neck PDFF, %	97	83.4 (76.5 to 87.9)	18	75.8 (69.2 to 82.8)	.**021**
Femoral neck aLUL, %	97	3.6 (3.2 to 4.2)	18	3.4 (3.1 to 3.6)	.11

Bold values represent statistically significant.

Values expressed as numbers (%), mean ± SD, or median (IQR).

Abbreviations: CTX, collagen type 1 cross-linked C-telopeptide; HRT, hormonal replacement therapy; hs-CRP, high-sensitivity C-reactive protein; IQR, interquartile range; PINP, procollagen type 1 N-terminal propeptide; PTH, parathyroid hormone; TIA, transient ischemic attack.

^*a*^Lumbar spine BMD measurements were not performed in 1 woman (vertebral fractures at L1, L2, and L3).

^*b*^Hip BMD measurements were not available in 3 women (bilateral hip arthroplasty).

^*c*^Hip PDFF measurements were not available in 7 women (bilateral hip arthroplasty, n = 3; bilateral hip osteonecrosis, n = 1; unacceptable quality of measurements, n = 3).

Patients with T2DM had significantly higher HbA1c (*P* < .001), glycemia (*P* < .001), and calcemia levels (*P* = .027) than patients without. PINP and CTX levels were lower in postmenopausal women with T2DM (*P* < .001 and *P* = .004, respectively). The areal BMD at the total hip, femoral neck, and lumbar spine was higher in postmenopausal women with T2DM (*P* < .05).

When imaging-based PDFFs were compared, femoral head, neck, and diaphysis PDFFs were lower in subjects with vs without T2DM (*P* < .05). No significant differences were observed at the lumbar spine between groups.

### Comparison of Hip and Lumbar Spine Imaging-Based PDFF in Patients With and Without T2DM

When femoral head, neck, and diaphysis PDFFs were compared after adjusting for age, BMI, and femoral neck BMD, no significant differences were found between patients with and without diabetes, except for the femoral head PDFF, which was lower in patients with T2DM (mean [SEM] 88.0% [0.7] vs 90.6% [0.3], *P* < .001) (Table 2).

**Table 2.** Adjusted mean imaging-based PDFF was assessed by presence of diabetes vs no diabetes

	N	Diabetes (N = 29)	N	No diabetes (N = 170)	Standardized difference (95% CI)	*P* value
PDFF femoral neck, %	29	79.9 ± 1.4	163^*a*^	82.3 ± 0.5	−0.29 (−0.63 to 0.05)	0.098
PDFF femoral head, %	29	88.0 ± 0.7	163^*a*^	90.6 ± 0.3	−0.63 (−0.99 to −0.26)	**0**.**008**
PDFF femoral diaphysis, %	29	79.1 ± 1.5	163^*a*^	80.9 ± 0.6	−0.19 (−0.52 to 0.15)	0.27
PDFF lumbar spine, %	29	61.2 ± 1.8	170	57.4 ± 0.7	0.37 (−0.003 to 0.75)	0.053

Bold values represent statistically significant.

Values are expressed as adjusted means ± standard error of the mean (SEM).

Standardized differences are presented with the 95% CI as the magnitude of between-group differences.

*P* values are adjusted for age, BMI, and femoral neck BMD (or lumbar spine BMD).

Abbreviations: BMD, bone mineral density; BMI, body mass index; PDFF, proton density fat fraction.

^*a*^Hip PDFF measurements were not available in 7 women (bilateral hip arthroplasty, n=3; bilateral hip osteonecrosis, n=1; unacceptable quality of measurements, n=3).

When imaging-based PDFFs were compared, after adjusting for age, BMI, and lumbar spine BMD, lumbar spine PDFF was higher in patients with T2DM compared to patients without, but the difference failed to achieve statistical significance (mean [SEM] 61.2% [1.8] vs 57.4% [0.7], *P* = .053).

### MRS-Based PDFF and aLUL at the L3 Vertebral Level and Femoral Neck

^1^H-MRS was performed at the L3 vertebral level in a subgroup of 113 patients. As illustrated in Table 3, no significant difference in PDFF at the L3 level was found between the groups after adjusting for age, BMI, and lumbar spine BMD. However, the aLUL at the L3 vertebrae was lower in patients with T2DM (n = 16) than in without (n = 97) (mean [SEM] 3.9% [0.1] vs 4.3% [0.1], *P* = .02).

**Table 3.** Comparison of PDFF and apparent lipid unsaturation level using ^1^H-MRS in patients with and without diabetes

	N	Diabetes	N	No diabetes	Standardized difference (95% CI)	*P* value
L3 PDFF, %	16	60.3 (2.9)	97	57.0 (1.1)	0.29 (−0.23 to 0.81)	.28
L3 aLUL, %	16	3.9 (0.1)	97	4.3 (0.1)	−0.63 (−1.16 to −0.1)	.**02**
Femoral neck PDFF, %	18	79.3 (2.2)	97	81.2 (0.8)	−0.18 (−0.61 to 0.27)	.44
Femoral neck aLUL, %	18	3.6 (0.2)	97	3.7 (0.1)	−0.10 (−0.58 to 0.38)	.67

Bold values represent statistically significant.

Values are expressed as adjusted means ± standard error of the mean (SEM).

Standardized differences are presented with the 95% CI as the magnitude of between-group differences.

*P* values are adjusted for age, BMI, and femoral neck BMD (or lumbar spine BMD).

Abbreviations: aLUL, apparent lipid unsaturation level; BMD, bone mineral density; BMI, body mass index; ^1^H-MRS, proton magnetic resonance spectroscopy; PDFF, proton density fat fraction.

^1^H-MRS was performed at the femoral neck in a subgroup of 115 participants. At the femoral neck, no significant differences in PDFF and aLUL were found between the groups after adjusting for age, BMI, and femoral neck BMD.

### Mediation Analyses

The total, direct, and indirect associations between T2DM and BMAT measurements (PDFF and aLUL) are presented in Table 4. Although the results are consistent with the previous analyses in terms of total association, no statistically significant mediating effect was found for both the presence of recent fracture and femoral neck BMD; therefore, it is not possible to conclude that these 2 previous characteristics have a mediating effect on the association between T2DM and BMAT. Regarding lumbar spine BMD, significant direct and indirect associations were observed between PDFF femoral head and T2DM (β = −2.15 [−3.67; −0.63], *P* = .0055 and β = −0.49 [−0.95; −0.03], *P* = .0386 respectively), indicating that 18.43% of the association between T2DM and PDFF femoral head was mediated by lumbar spine BMD.

**Table 4.** Adjusted direct and indirect associations of presence of type 2 diabetes mellitus with (i) imaging-based PDFF; and (ii) PDFF and apparent lipid unsaturation level using ^1^H-MRS, mediated by the presence of recent fracture or BMD (femoral neck or lumbar spine)

Imaging-based PDFF	Presence of recent fracture		Femoral neck BMD		Lumbar spine BMD	
	Beta (95% CI)	*P* value	Beta (95% CI)	*P* value	Beta (95% CI)	*P* value
PDFF femoral neck (%)						
Total association	−2.5 (−5.36;0.35)	.086	−2.5 (−5.35;0.36)	.0867	−2.45 (−5.34;0.44)	.0968
Direct association	−2.46 (−5.31;0.38)	.0898	−2.49 (−5.38;0.4)	.0916	−1.49 (−4.43;1.44)	.3179
Indirect association	−0.04 (−0.31;0.23)	.7736	−0.01 (−0.45;0.44)	.9736	−0.96 (−1.85;−0.06)	.0368
Proportion mediated (%)	1.57	NA	0.3	NA	39.01	NA
PDFF femoral head (%)						
Total association	−2.64 (−4.12;−1.16)	.0005	−2.63 (−4.11;−1.16)	.0005	−2.64 (−4.14;−1.14)	.0005
Direct association	−2.61 (−4.08;−1.14)	.7697	−2.63 (−4.13;−1.13)	.0006	−2.15 (−3.67;−0.63)	.0055
Indirect association	−0.03 (−0.24;0.17)	.7679	0 (−0.23;0.23)	.9743	−0.49 (−0.95;−0.03)	.0386
Proportion mediated (%)	1.16	NA	0.14	NA	18.47	NA
PDFF femoral diaphysis (%)						
Total association	−1.95 (−5.14;1.24)	.2301	−1.95 (−5.13;1.24)	.2308	−1.86 (−5.08;1.36)	.2573
Direct association	−1.93 (−5.12;1.25)	.234	−1.83 (−5.05;1.39)	.2651	−0.88 (−4.16;2.39)	.5974
Indirect association	−0.02 (−0.15;0.12)	.804	−0.12 (−0.62;0.39)	.6561	−0.98 (−1.95;−0.01)	.0487
Proportion mediated (%)	0.88	NA	5.93	NA	52.6	NA
PDFF lumbar spine (%)						
Total association	2.69 (−1.02;6.4)	.1549	2.77 (−0.96;6.49)	.1451	2.91 (−0.84;6.66)	.128
Direct association	2.67 (−1.04;6.38)	.1578	3.13 (−0.62;6.87)	.102	3.84 (0.03;7.65)	.0483
Indirect association	0.02 (−0.16;0.2)	.8206	−0.36 (−1.01;0.29)	.2756	−0.93 (−1.96;0.1)	.078
Proportion mediated (%)	0.78	NA	−12.99	NA	−31.89	NA
^1^ **H-MRS PDFF and aLUL**						
PDFF L3 (%)						
Total association	2.39 (−3.47;8.24)	.424	2.4 (−3.48;8.29)	.4237	2.96 (−2.98;8.91)	.3287
Direct association	2.35 (−3.52;8.22)	.4326	2.5 (−3.38;8.38)	.4042	3.36 (−2.59;9.32)	.2678
Indirect association	0.04 (−0.41;0.49)	.8661	−0.1 (−0.59;0.39)	.6922	−0.4 (−1.34;0.54)	.4018
Proportion mediated (%)	1.61	NA	−4.11	NA	−13.57	NA
L3 aLUL (%)						
Total association	−0.18 (−0.51;0.15)	.2779	−0.18 (−0.51;0.15)	.277	−0.34 (−0.63;−0.05)	.0204
Direct association	−0.19 (−0.52;0.14)	.2492	−0.18 (−0.51;0.15)	.2798	−0.35 (−0.64;−0.06)	.0173
Indirect association	0.01 (−0.03;0.05)	.5616	0 (−0.02;0.01)	.9066	0.01 (−0.03;0.05)	.5804
Proportion mediated (%)	−6.07	NA	0.49	NA	−3.11	NA
PDFF Femoral neck (%)						
Total association	−2.82 (−7.09;1.46)	.197	−2.81 (−7.09;1.46)	.1974	−2.54 (−6.89;1.81)	.2519
Direct association	−2.72 (−6.97;1.52)	.2088	−2.95 (−7.3;1.39)	.183	−1.85 (−6.39;2.69)	.4241
Indirect association	−0.09 (−0.66;0.47)	.7443	0.14 (−0.67;0.96)	.7328	−0.69 (−2.11;0.73)	.3399
Proportion mediated (%)	3.34	NA	−5.05	NA	27.19	NA
Femoral neck aLUL (%)						
Total association	−0.18 (−0.52;0.16)	.3101	−0.18 (−0.52;0.16)	.3085	−0.24 (−0.58;0.11)	.1785
Direct association	−0.19 (−0.52;0.15)	.2799	−0.08 (−0.42;0.25)	.6317	−0.08 (−0.42;0.27)	.6642
Indirect association	0.01 (−0.05;0.06)	.7418	−0.1 (−0.21;0.02)	.093	−0.16 (−0.3;−0.02)	.0254
Proportion mediated (%)	−5.2	NA	53.91	NA	67.43	NA

Adjusted for age and BMI.

Abbreviations: aLUL, apparent lipid unsaturation level; BMD, bone mineral density; BMI, body mass index; ^1^H-MRS, proton magnetic resonance spectroscopy; NA, not applicable; PDFF, proton density fat fraction.

### Correlations Between Lumbar Spine and Hip Imaging- and MRS-Based PDFF and HbA1c

Table 5 shows the correlations between imaging- and MRS-based PDFF and HbA1c. No significant correlations were found between the femoral head, neck, and diaphysis imaging-based PDFF and HbA1c. A small positive correlation was found between lumbar spine PDFF and HbA1c in all patients (n = 198) and patients without T2DM (n = 169) (all: *R* = 0.17, *P* = .016; no T2DM: *R* = 0.19, *P* = .012). No significant correlations were found between the femoral neck, and L3 MRS-based PDFF and HbA1c.

**Table 5.** Correlations between lumbar spine and hip imaging- and MRS-based PDFF and HbA1c

	All N = 198	Diabetes N = 29	No diabetes N = 169
Lumbar spine PDFF, %*^a^*	***R* = 0.17 (−0.03 to 0.30)** ***P* = .016**	*R* = −0.34 (−0.63 to 0.03) *P* = .069	***R* = 0.19 (0.04 to 0.33)** ***P* = .012**
Femoral head PDFF, %*^b^*	*R* = −0.09 (−0.23 to 0.05)) *P* = .20	*R* = −0.12 (−0.46 to 0.26) *P* = .54	*R* = 0.10 (−0.06 to 0.25) *P* = .23
Femoral neck PDFF, %*^b^*	*R* = −0.10 (−0.23 to 0.05) *P* = .19	*R* = −0.27 (−0.57 to 0.12) *P* = .16	*R* = 0.04 (−0.11 to 0.20) *P* = .59
Femoral diaphysis PDFF, %*^b^*	*R* = −0.07 (−0.27 to 0.08) *P* = .37	*R* = −0.19 (−0.52 to 0.19) *P* = .31	*R* = 0.06 (−0.10 to 0.21) *P* = .47
L3 PDFF, %*^c^* (MRS)	*R* = 0.11 (−0.08 to 0.29) *P* = .25	*R* = −0.34 (−0.71 to 0.20) *P* = .20	*R* = 0.10 (−0.10 to 0.30) *P* = .33
Femoral neck PDFF, %*^d^* (MRS)	*R* = −0.08 (−0.26 to 0.11) *P* = .41	*R* = −0.04 (−0.50 to 0.43) *P* = .87	*R* = 0.08 (−0.13 to 0.27) *P* = .47

Bold values represent statistically significant.

Spearman correlation coefficients are expressed with the 95% CI.

Abbreviations: HbA1c, glycated hemoglobin; MRS, magnetic resonance spectroscopy; PDFF, proton density fat fraction (imaging-based).

^*a*^n = 198; Lumbar spine PDFF measurements were not performed in 1 woman (vertebral fractures at L1, L2, and L3).

^*b*^n = 192; Hip PDFF measurements were not available in 7 women (bilateral hip arthroplasty, n = 3; bilateral hip osteonecrosis, n = 1; unacceptable quality of measurements, n = 3).

^*c*^n = 113.

^*d*^n = 115.

## Discussion

### Main Findings

Upon comparing the lumbar spine imaging-based PDFF between diabetic and nondiabetic subjects, no statistically significant difference was observed, although the difference approached the limit of significance. Another finding was that a lower femoral head PDFF derived from water-fat imaging was associated with diabetic status in postmenopausal women, independent of age, BMI, and femoral neck BMD. PDFF measurements at the proximal femur rather than at the lumbar spine may be useful for assessing the relationship between marrow adiposity and diabetic status in this population. In accordance with previous findings, ^1^H-MRS vertebral measurements found that diabetes was associated with a lower unsaturation level, while also supporting the use of marrow adiposity composition to assess its connection.

### Comparison With Other Studies

The examination and understanding of the relationship between marrow adiposity and T2DM has recently emerged as an exciting area of research. Bone marrow adiposity may alter or may be altered by metabolic conditions, including T2DM. In preclinical studies, distinct mouse models of diabetes induction have demonstrated similar bone phenotypes marked by significant trabecular bone loss and increased BMAT [13, 14]. Only a few studies have investigated marrow adiposity content and composition in patients with T2DM compared to nondiabetic patients [12, 15-18]. Intriguingly, this relationship may differ according to sex, measurement site, and the methodology used to evaluate marrow adiposity [12, 15-18].

A case-control study was conducted by Baum et al on 26 postmenopausal women, comprising 13 nondiabetic and 13 age- and BMI-matched diabetic subjects [15]. The study included analyses of blood parameters, such as plasma glucose and HbA1c levels, as well as measurements of bone marrow fat fraction (BMFF) and unsaturation levels using ^1^H-MRS (L1-L3). The findings revealed that the mean vertebral BMFF was similar between women with diabetes and healthy controls (69.3 ± 7.5% vs 67.5 ± 6.1%). However, the mean unsaturation level was significantly lower in the diabetic group than in the healthy controls (6.7 ± 1.0% vs 7.9 ± 1.6%; *P* < .05). Furthermore, patients with poor glycemic control (HbA1c > 7%, n = 8) in the diabetic group demonstrated a significantly higher mean BMFF than those with good glycemic control (HbA1c < 7%, n = 5: 73.5% ± 5.6% vs 64.1% ± 6.9%, *P* < .05) [15]. Similar results were reported by Patsch et al [16], who found no association between diabetes status and marrow adiposity in the lumbar spine using ^1^H-MRS in a cohort of 69 postmenopausal women (mean age, 63 ± 5 years; BMI range, 18-37 kg/m^2^), whereas diabetes was associated with a −1.3% lower unsaturation level (95% CI, −2.3% to −0.2%; *P* = .018) after adjusting for age, race, and spine BMD using quantitative computed tomography. In accordance with previous studies, we found no association between lumbar spine PDFF and diabetes status using either water-fat imaging- or MRS-based measurements, whereas diabetes was associated with lower unsaturation levels at this measurement site.

Sheu et al [17] found that mean vertebral BMFF using ^1^H-MRS was higher in men with diabetes (n = 38) than those without (n = 118) (59.2 ± 3.5% vs 54.8 ± 3.3%, *P* = .036) after adjusting for age, race, and BMI, but this result did not remain significant after excluding 2 men receiving thiazolidinediones [17]. Another recent study specifically performed sex-stratified analyses to evaluate alterations in marrow adiposity in patients with diabetes [22]. ^1^H-MRS scans of the spine and distal tibia were performed in patients with diabetes and in normoglycemic controls (n = 39/37), including 34 males (n = 18/16). At the spine but not at the distal tibia, the PDFF and unsaturation levels were lower in male diabetic subjects than in male controls in an age-adjusted model [22]. No significant differences in PDFF and unsaturation levels were found in female individuals between the groups, either at the spine or distal tibia [22].

A cross-sectional study was conducted by Yu et al on 21 subjects with morbid obesity (18 women; mean age, 49 ± 11 years; mean BMI 43.9 ± 5.4 kg/m^2^) prior to bariatric surgery, comprising 13 nondiabetic and 8 diabetic subjects [18]. The study included analyses of HbA1c levels as well as measurements of BMFF and unsaturation levels using ^1^H-MRS at the vertebrae, femoral diaphysis, and metaphysis. The findings revealed that the lipid/water ratio was higher in diabetic subjects than in nondiabetic subjects at the vertebrae and femoral metaphysis (*P* ≤ .04). Furthermore, the lipid unsaturation index (UI) was significantly lower at the femoral diaphysis in diabetic subjects (*P* = .03) [18]. Despite the small number of diabetic subjects, this study is interesting because of the marrow adiposity assessment at both the spine and femur, highlighting both an increase in the content at these 2 sites and a lower level of unsaturation at the femoral diaphysis. However, this specific population (morbid obesity prior to bariatric surgery) did not allow us to extrapolate these results to the general population. Unlike this study, we found a *lower* femoral (head) PDFF in diabetic subjects without any association with the level of unsaturation at this site; however, assessment of marrow adiposity content and composition was performed in our study at the proximal femur (head, neck, and diaphysis) in postmenopausal women, which limits the comparison. Furthermore, findings on *lower* femoral (head) PDFF in diabetic subjects should be interpreted cautiously and need to be replicated in further studies given that the effect size was moderate.

A recent study using bone histology/histomorphometry rather than MRI improved our understanding of the relationship between marrow adiposity and T2DM [12]. Indeed, bone histomorphometry data from 26 premenopausal women with diabetes suggested that increased BMAT occurs through adipocyte hyperplasia and hypertrophy. Indeed, adipocyte number (Ad.N), perimeter (Ad.Pm), and total area of adipocytes (Ad.Ar) were higher in the poor control group (HbA1c > 7%, n = 16) than in the good control group (HbA1c < 7%, n = 10) (all *P* = .04) [12]. Further studies using bone histology/histomorphometry are needed in specific populations, such as men and postmenopausal women, and in a control group to compare diabetic and nondiabetic subjects.

Given that there are alterations in BMAT measurements in the case of recent fractures [19] and T2DM, it seems relevant to investigate whether the presence of recent fractures was a mediator of the association between T2DM and BMAT measurements. No statistically significant mediating effect was found, leading to the conclusion that the presence of a recent fracture has no mediating effect on the association between T2DM and BMAT measurements. Regarding BMD, the association between T2DM and PDFF femoral head was weakly mediated by lumbar spine BMD, which should be interpreted cautiously given the exploratory nature of these analyses.

Furthermore, in women with T2DM, higher HbA1c levels were associated with higher marrow adiposity content, suggesting that marrow adiposity may influence or may be influenced by glucose metabolism and glycemic control [12, 15]. Moreover, positron emission tomography (PET) scanning suggested that marrow adiposity is a major source of basal glucose uptake [23]. In our study, weak positive correlations between the PDFFs and HbA1c levels were found only at the lumbar spine.

### Strengths and Weakness

Our study has several strengths. This ancillary study of the ADIMOS cohort yielded an original sample comprising postmenopausal women with or without T2DM. Therefore, this population is homogeneous, limiting biases secondary to estrogen contribution. Chemical shift encoding-based WFI was performed considering T1 bias and T2* decay, providing an accurate PDFF at the lumbar spine and proximal femur. Despite the superior accuracy of ^1^H-MRS, the use of chemical shift-based WFI allowed straightforward measurements that were achievable in most MR clinical systems, while maintaining sufficient accuracy. Finally, the DXA and MRI examinations were performed using the same machines with the same parameters, reinforcing the comparability of the subjects’ data. However, this study has several limitations. No sample size was preliminarily calculated, and this study was probably not adequately powered for comparison between diabetic and nondiabetic patients. The cross-sectional design prevented us from assessing the temporal association between the PDFF and glucose metabolism. Another limitation is the choice of women with osteoarthritis as the control group. Moreover, postmenopausal women with T2DM were relatively well controlled, which may contribute to the lack of differences. Additionally, our findings apply only to postmenopausal women and cannot be generalized to men and younger age groups.

## Conclusions

Proximal femur imaging-based PDFF might be a noninvasive biomarker that can differentiate between postmenopausal women with and without diabetes. Moreover, diabetes is associated with a lower level of unsaturation at the L3 vertebral level. We recommend that future studies investigating BMAT and diabetes report measurements of marrow adiposity content and composition at both the proximal femur PDFF and lumbar spine PDFF. Additional research is needed to further define the role of marrow adiposity in diabetic skeletal fragility and determine whether marrow adiposity is a therapeutic target.

## Data Availability

The data supporting the findings of this study are available from the corresponding author upon reasonable request.

## Abbreviations

AGE: advanced glycation end product
aLUL: apparent lipid unsaturation level
BMAT: bone marrow adipose tissue
BMD: bone mineral density
BMFF: bone marrow fat fraction
BMI: body mass index
CTX: collagen type 1 cross-linked C-telopeptide
DXA: dual-energy x-ray absorptiometry
^1^H-MRS: proton magnetic resonance
HbA1c: glycated hemoglobin; spectroscopy
MRI: magnetic resonance imaging
PDFF: proton density fat fraction
PINP: procollagen type I amino-terminal propeptide
SEM: standard error of the mean
T2DM: type 2 diabetes mellitus
WFI: water-fat separation imaging
